# Rehabilitation Nutrition in Older People

**DOI:** 10.3390/nu15081893

**Published:** 2023-04-14

**Authors:** Yoji Kokura, Ryo Momosaki

**Affiliations:** 1 Department of Nutritional Management, Keiju Hatogaoka Integrated Facility for Medical and Long-Term Care, Hosu 927-0023, Japan; 2 Department of Rehabilitation Medicine, Mie University Graduate School of Medicine, Tsu 514-8507, Japan

Rehabilitation nutrition is expected to help treat frailty, undernutrition, and sarcopenia. It is defined as (i) holistically evaluating the presence and causes of nutritional disorders, sarcopenia, and excess or deficient nutrient intake using the International Classification of Functioning, Disability, and Health; (ii) conducting rehabilitation nutrition diagnosis and goal setting; and (iii) inducing the highest levels of body functions, activities, participation, and quality of life in patients by improving their nutritional status, sarcopenia, and frailty [1,2]. Previous studies have reported that rehabilitation nutrition intervention can help improve activities of daily living (ADL) [3,4]. Additionally, high-quality rehabilitation nutrition can be provided through a rehabilitation nutrition care process [1,2]. This care process includes the assessment and diagnostic reasoning [5], diagnosis, goal setting [6], intervention, and monitoring of rehabilitation nutrition [7,8,9]. However, clinical practice guidelines for rehabilitation nutrition have weakly recommended enhanced nutritional care for rehabilitation patients with cerebrovascular disease, hip fracture, cancer, and acute illnesses [10]. Therefore, the expected benefits of rehabilitation nutrition remain controversial.

Herein, we discuss two review articles and eight original articles on rehabilitation nutrition. Shirai et al. conducted a narrative review of the prevalence of the relationship between nutrition-related problems and falls among patients undergoing hemodialysis (HD) [11]. Their literature search revealed several studies showing the association of frailty and undernutrition with an increased risk of falls in patients undergoing HD. These falls were found to be caused by nutritional problems, such as iatrogenic and noniatroenic factors, and nutritional therapy may help prevent falls. A narrative review by Okamura et al. summarized the current evidence and interventions related to rehabilitation nutrition for cachexia and protein–energy wasting (PEW) in patients with chronic kidney disease [12]. Although nutritional management and exercise therapy were independently effective for cachexia and PEW, the combined effect of nutrition and exercise interventions remains unclear. We conducted a cross-sectional study to investigate the prevalence of malnutrition diagnosed based on the Global Leadership Initiative on Malnutrition (GLIM) criteria and its association with ADL in an older residents’ integrated facility for medical and long-term care [13]. We revealed that the prevalence of mild malnutrition was 29%, and 18% of the cases were severe. Multivariate analyses of the Barthel scale/index (BI) score after adjusting for potential confounders revealed that mild and severe malnutrition were independently associated with the BI score. Nishioka et al. examined the association between the presence of sarcopenia and poor recovery of swallowing function in poststroke patients with severe deglutition disorder in convalescent rehabilitation hospitals in Japan [14]. This multicenter cohort study revealed that sarcopenia was negatively associated with the recovery of swallowing function in patients with stroke and was independent of energy intake and rehabilitation duration. Matsui et al. reported that high visceral fat mass caused severe complications but improved long-term prognosis after radical gastrectomy in patients with advanced gastric cancer [15].

Furthermore, a cross-sectional study conducted by Togashi et al. revealed that although the area under the curve of body mass index (BMI) for sarcopenic dysphagia diagnosis was approximately 0.6, the BMI of <20.0 kg/m^2^ might be a predictor for sarcopenic dysphagia [16]. They revealed that in clinical settings if patients with dysphagia had a BMI of <20.0 kg/m^2^, sarcopenic dysphagia should be suspected immediately after admission. Abe et al. examined the additive effect of energy intake and rehabilitation time on ADL improvement in patients with acute stroke and sarcopenia [17] and demonstrated that a combination of high energy intake and sufficient rehabilitation time was associated with ADL improvement. Shirai et al. investigated and validated whether Asian BMI cutoff values can accurately predict 30-day in-hospital mortality, length of hospital stay, and 90-day readmission outcomes for patients with acute chronic obstructive pulmonary disease exacerbations [18]. They demonstrated that the severity of low BMI, based on the Asian BMI cutoff values of the GLIM criteria, was independently associated with 30-day in-hospital mortality. A cross-sectional study by Nagano et al. determined the predictive value of temporal muscle thickness (TMT) via computed tomography to detect sarcopenia after acute stroke in older patients [19]. They demonstrated that the TMT cutoff values for identifying sarcopenia and low skeletal muscle index were the same: 3.83 mm for men and 2.78 mm for women. Finally, Abe et al. conducted a retrospective cohort study to examine the effects of undernutrition on swallowing function and ADL in hospitalized patients [20]. Multivariable analysis revealed that undernutrition is associated with reduced improvement in swallowing function and ADL.

These articles have focused on the assessment, intervention, and prediction outcomes related to patients in rehabilitation. However, no report on the rehabilitation nutrition care process has been published to date; thus, future studies should address this issue. Further scientific evidence on rehabilitation nutrition will help overcome the problems associated with frailty, sarcopenia, and malnutrition.
